# Supplementary material for: A Retrospective Study of the Proportion of Women at High and Low Risk of Intrauterine Infection Meeting Sepsis Criteria
Source: Microorganisms. 2021 Dec 31;10(1):82. doi: 10.3390/microorganisms10010082 (PMC8779684; doi:10.3390/microorganisms10010082)
Supplement: Supplementary file 1 [file microorganisms-10-00082-s001.zip › microorganisms-1489585-supplementary.pdf]

Table S1. Comparison of subjects included in study versus subjects excluded due to missing data.

	PPROM			CONTROL		
	Included cases *n* = 453 (%)	Missing data *n* = 132 (%)	p	Included cases *n* = 2004 (%)	Missing data *n* = 1155 (%)	p
Maternal age (mean ± CI)	31.5	31.2	0.650	33.8	33.4	0.030
>35 year	127 (28)	36 (27.3)	0.860	749 (37.4)	420 (36.4)	0.552
Completed secondary education	411 (90.7)	127 (96.2)	0.100	1934 (96.5)	1123 (97.2)	0.534
Gestation number (mean ± CI)	3.2	3.2	0.890	3.9	3.7	0.039
Previous cesareans (mean ± CI)	0.4	0.4	0.930	1.1	0.9	0.001
In vitro fertilization	126 (27.8)	31 (23.8)	0.320	319 (15.9)	170 (14.7)	0.369
Gestational diabetes	46 (10.2)	10 (7.6)	0.380	269 (13.4)	154 (13.3)	0.943
Hypertension	18 (4)	8 (6.1)	0.310	68 (3.4)	59 (5.1)	0.022

PPROM: preterm premature rupture of membranes; CI: confidence interval.

Table S2. Subjects with missing vital signs.

Parameter		PPROM *n* = 585 (%)	Control *n* = 3159 (%)
Admission	MAP	57 (9.7)	129 (4.1)
	Pulse	67 (11.5)	123 (3.9)
	Temperature	105 (17.9)	322 (10.2)
POD 0	MAP	3 (0.5)	345 (10.9)
	Pulse	2 (0.3)	286 (9.1)
	Temperature	101 (17.3)	2629 (83.2)
POD 1	MAP	2 (0.3)	247 (7.8)
	Pulse	2 (0.3)	41 (1.3)
	Temperature	4 (0.7)	127 (4)
POD 2	MAP	9 (1.5)	239 (7.6)
	Pulse	12 (2.1)	183 (5.8)
	Temperature	4 (0.7)	76 (2.4)

PPROM: preterm premature rupture of membranes; POD: post-operation day; MAP: mean arterial pressure.

Table S3. Sensitivity analysis – urinary cultures. PPROM: preterm premature rupture of membranes.

	**PPROM *n* = 453 (%)**	**Control *n* = 2004 (%)**	*p*-value
Positive cultures if missing are positive	64 (14.1)	60 (3)	<0.001
Positive cultures if missing are negative	25 (5.5)	60 (3)	0.008
